# Signals of Historical Interlocus Gene Conversion in Human Segmental Duplications

**DOI:** 10.1371/journal.pone.0075949

**Published:** 2013-10-04

**Authors:** Beth L. Dumont, Evan E. Eichler

**Affiliations:** 1 Department of Genome Sciences, University of Washington, Seattle, Washington, United States of America; 2 Howard Hughes Medical Institute, Seattle, Washington, United States of America; Louisiana State University, United States of America

## Abstract

Standard methods of DNA sequence analysis assume that sequences evolve independently, yet this assumption may not be appropriate for segmental duplications that exchange variants via interlocus gene conversion (IGC). Here, we use high quality multiple sequence alignments from well-annotated segmental duplications to systematically identify IGC signals in the human reference genome. Our analysis combines two complementary methods: (i) a paralog quartet method that uses DNA sequence simulations to identify a statistical excess of sites consistent with inter-paralog exchange, and (ii) the alignment-based method implemented in the GENECONV program. One-quarter (25.4%) of the paralog families in our analysis harbor clear IGC signals by the quartet approach. Using GENECONV, we identify 1477 gene conversion tracks that cumulatively span 1.54 Mb of the genome. Our analyses confirm the previously reported high rates of IGC in subtelomeric regions and Y-chromosome palindromes, and identify multiple novel IGC hotspots, including the pregnancy specific glycoproteins and the neuroblastoma breakpoint gene families. Although the duplication history of a paralog family is described by a single tree, we show that IGC has introduced incredible site-to-site variation in the evolutionary relationships among paralogs in the human genome. Our findings indicate that IGC has left significant footprints in patterns of sequence diversity across segmental duplications in the human genome, out-pacing the contributions of single base mutation by orders of magnitude. Collectively, the IGC signals we report comprise a catalog that will provide a critical reference for interpreting observed patterns of DNA sequence variation across duplicated genomic regions, including targets of recent adaptive evolution in humans.

## Introduction

The ancestral lineage leading to humans and great apes experienced a surge in the rate of genomic duplication and deletion [1]–[5]. This sudden burst of large-scale rearrangement contributed to an exceptional human genomic architecture characterized by ∼166 Mb of paralogous sequences with >90% sequence identity, including over 95 Mb of sequences with >98% identity [1]. These duplicated regions, termed segmental duplications (SDs), provide abundant templates for non-allelic homologous recombination (NAHR) during double-strand break repair. Ectopic crossing over, one mechanism of NAHR, can generate large deletions, duplications, and translocations. These rearrangements impose an enormous disease burden on human populations, and contribute to species-specific genome structural innovations [6]–[9].

A second mechanism of NAHR is non-crossover associated interlocus gene conversion (IGC). During IGC, sequence from a donor SD is copied and pasted into the paralogous position of the acceptor SD. Unlike ectopic crossovers, IGC events do not introduce changes in copy number. In addition, the converted sequence is short, usually <500 bp [10]–[13], although rare long-track (>10 kb) gene conversion events have been identified in some organisms [14]–[16]. The genetic signature of an IGC event is subtle, and escapes detection by standard methods for assaying structural variation, such as array comparative genome hybridization.

Despite its limited footprint, both theoretical and empirical studies suggest that IGC is an important determinant of sequence variation in duplicated regions. IGC can transfer variants that arise on the genetic background of one locus to the novel genetic context of a paralogous position, thereby enabling the spread of new variants between duplicated sequences [17]–[19]. In this manner, IGC can increase within locus haplotype diversity while simultaneously decreasing sequence diversity between loci, contributing to their concerted evolution [20]. This mechanism may enable ancient duplicate sequences to maintain high sequence identity [21], and may even facilitate the retention of functional similarity between paralogs [22].

IGC also bears on the genetic analysis of duplicated regions. Models of evolutionary divergence assume that sequences evolve independently, but duplicated sequences actively exchanging variants via IGC violate this assumption. By homogenizing paralogous sequences, IGC can slow the apparent rate of molecular evolution between paralogs and invalidate the molecular clock hypothesis. Paralogous sequence divergence times are therefore likely to be underestimated in the face of IGC. These points may deserve particular consideration in analyses of duplicated sequences in the human genome. Recently duplicated genes are enriched for signals of positive selection [23] and present alluring candidates contributing to the genetic basis of uniquely human traits [3]; [24]–[26]. However, if the confounding effects of IGC are not considered, standard methods of evolutionary sequence analysis may yield misleading inferences about the selective regimes at work and the true evolutionary age of duplication events.

Focused gene family studies and genome-wide analyses have shed light on the distribution of IGC in mammalian genomes. IGC is most frequent between high-identity tandem duplicates [27]–[30], and plays a key role in shuttling disease-causing variants between pseudogenes and functionally active paralogs [31]. Several IGC “hotspots” – including the opsins [32], olfactory receptors [33], ribosomal RNA genes [34], and subtelomeric repeats [35] – have been identified. Nonetheless, methods for detecting IGC have very low power for high identity regions with low to moderate rates of historical IGC [36]. Given the enrichment of young, highly identical SDs in primate genomes, our current understanding of IGC and its effects on duplicate sequence evolution is incomplete.

One barrier to a more comprehensive understanding of the frequency and distribution of IGC is the difficulty of properly annotating and assembling duplicated genomic regions. High-identity SDs are often mis-placed or collapsed to a single locus in draft genomes. The active homogenization of duplicated sequences by IGC may further exacerbate this challenge. However, since the initial release of the human genome, SDs have been the overwhelming focus of targeted re-sequencing, BAC-based assembly, and manual curation [37]. As a consequence, the current freeze of the human genome is nearly complete with respect to these complex duplicated regions. Here, we leverage these recent advances in the quality and completeness of the human reference genome to develop the most comprehensive catalog of IGC events in the human genome to date. We report clear IGC signals across upward of 4% of the total duplicated sequence surveyed, including several previously uncharacterized IGC hotspots. We show that these detected signals recapitulate known features of the genomic distribution of IGC and key properties of IGC tracks. Our analyses indicate that IGC has introduced incredible levels of fine-scale variation in the evolutionary relationships between SDs in the human genome, a result that carries important considerations for sequence-based evolutionary inference across these regions.

## Methods

### Multiple Sequence Alignment and Tree Construction

We used the *genomicSuperDups* table from the UCSC Table Browser to identify 1419 families of ≥4 paralogous sequences (mean number of sequences per family = 8.4) in the human reference genome (GRCh37). We limited our focus to large SDs >10 kb (mean sequence length = 34.7 kb) to ensure an adequate number of informative sites in tests of historical recombination (see below). Owing to the hierarchical, nested organization of SDs across the human genome, there is considerable sequence redundancy between SD families. Collectively, sequences within these families span 52.4 Mb of the human genome (1.7%) with each family surveying an average 29.4 kb of duplicated sequence not present in any other SD family. This initial dataset includes sequences overlapping 712 genes, with 205 genes fully contained within analyzed sequences.

We constructed a high quality multiple sequence alignment for each SD family using the *L-INS-i* algorithm implemented in *mafft* (v. 6.951) [38]. Alignments were trimmed to remove terminal regions of non-alignment and all gaps. We subsequently removed any sequences with <88% pairwise sequence identity to any other sequence in the alignment. Below this level of sequence identity, the rate of IGC is likely negligible [12]. Sequences that reduced overall alignment length by a factor >0.25 through the introduction of a large number of gap sites were excluded, and families with <10 kb aligned, ungapped sequence or fewer than four sequences were removed from the analysis (n = 83). The remaining 1336 SD families had an average of 7.7 paralogous sequences with 95.8% sequence identity across 18.8 kb of high quality aligned sequence.

To infer the evolutionary relationships between paralogous sequences within each SD family, we constructed maximum likelihood unrooted trees using PhyML (v3.0) [39]. Nucleotide evolution was modeled as a general time reversible process with gamma distributed rate variation across four rate classes and a proportion of invariant sites estimated by maximum likelihood [40]. To ensure high confidence in the resulting tree topology, we pruned the subset of trees with nodes supported by less than 88% of bootstrap replicates. Specifically, we randomly dropped one sequence stemming from the node with the lowest bootstrap support and reconstructed the tree on the reduced sequence alignment. This process was reiterated until all nodes on the tree were supported by ≥88% of bootstrap replicates, or until the alignment was reduced to fewer than four sequences. Fifty-six SD families were eliminated by this criterion (**Table S1)**. We excluded an additional 34 SD families for which neighbor-joining tree topologies based on an F84 DNA distance matrix were not equivalent to the maximum likelihood topologies [41]. The final set of 1246 SD families includes 38.9 Mb of sequence covering 617 genes.

### Quartet Analysis of Interlocus Recombination

**Figure 1:**
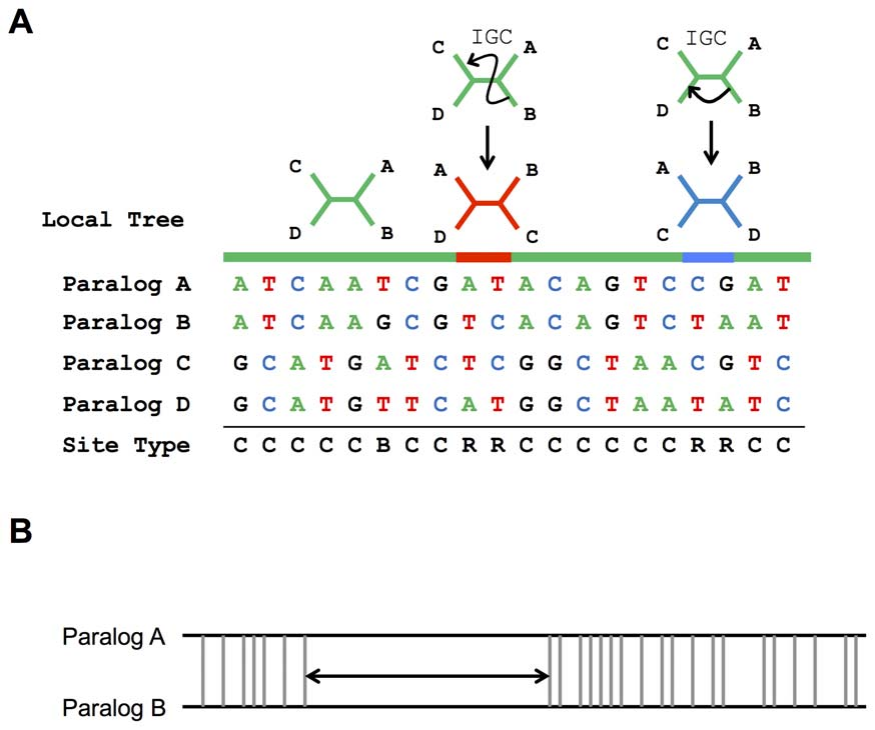
Methods for identifying historical IGC. (**A**) Local switches in tree topology can be driven by interlocus gene conversion (IGC). Bases at informative sites are shown for 4 paralogous sequences. The duplication history of these 4 sequences is captured by the unrooted green tree, which places paralogs A and B (C and D) as most closely related. Informative sites that are consistent with this topology are designated as concordant sites. Interlocus recombination can introduce sites that are discordant with this primary tree. Two interlocus gene conversion (IGC) events are illustrated, each giving rise to a small patch of reticulate sites supporting an alternative evolutionary relationship between paralogs (the red and blue trees). A third class of sites, bimutational sites, are sites of obligate double mutation at paralogous positions. (**B**) GENECONV scans paralog alignments to identify pairs of sequences with longer than expected tracks of perfect identity, conditioning the search on the overall pattern of variable sites in the alignment.

Although the evolutionary history of each sequence family is summarized by a single dominant tree topology (which presumably mirrors the duplication history of the sequences), individual sites within the alignment may be discordant with the tree. As illustrated in **Figure 1A**, non-allelic recombination between two sequences can give rise to informative sites that support an alternative tree topology. Non-allelic crossover events will introduce large, contiguous blocks of variable sites in the alignment that support an alternative tree topology. Gene conversion between non-allelic sequences can create short clusters of variable sites that are inconsistent with the dominant tree (**Figure** **1A**). The inter-digitation of IGC tracks, ectopic crossovers, and more complex rearrangements (*e.g.*, microhomology-mediated break-induced replication (MMBIR)) over time will generate complex, interwoven switches in the evolutionary history across a multiple sequence alignment [42]. In keeping with the vocabulary of Jackson et al. [27], we refer to sites inconsistent with the tree as “*R*eticulate sites” (R sites). In addition to interlocus recombination, R sites can also arise from independent mutation to the same nucleotide at paralogous positions. Most double mutation events, however, are expected to result in three bases at a given site in the alignment. We refer to these sites of obligate double mutation as “*B*imutational Sites” (B sites). Informative sites consistent with the dominant tree topology are classified as “*C*oncordant sites” (C sites).

An SD family with *N* paralogous sequences is comprised of 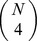 four-sequence sub-alignments, or quartets. For each sequence quartet within a SD family, we classified all informative sites as concordant, reticulate, or bimutational. The total number of R sites in the full alignment is the number of sites (or columns in the alignment) that are reticulate in ≥1 sequence quartet. We bootstrapped the columns of the alignment 100 times and calculated the number of R sites across each bootstrapped alignment to derive approximate 95% confidence intervals for the observed number of R sites.

We evaluate the hypothesis of historical IGC among sequences in a given quartet by comparing the observed number of R sites to the number expected in the absence of interlocus exchange. This null expectation was derived by simulating 100 sequence datasets along the ML tree using Seq-Gen (v1.3.3) [43]. We used the proportion of invariant sites, four classes of evolutionary rates, empirical nucleotide frequencies, and transition probabilities estimated from the actual data to guide the simulations (**Table S2**).

Approximate *P*-values for comparisons of the observed and expected numbers of R sites were computed using a strategy that accounts for uncertainty in both values. First, each draw from the bootstrap distribution of observed R sites was paired with a draw from the simulated distribution of R sites (n = 100 paired values). If the simulated distribution is a good approximation to the observed distribution, the difference between these values should be zero on average. On the other hand, if more R sites are observed than expected according to our simulation, the difference between the observed and simulated R site counts should be greater than zero. *P*-values were calculated as the fraction of 100 paired observations for which the simulated number of R sites was greater than the corresponding value from the bootstrap distribution.

### GENECONV Analysis

We used the program GENECONV to confirm the general trends documented by the quartet method and identify IGC events that may have gone undetected by the method [44]. GENECONV uses overall patterns of variation among sequences within an alignment to identify sequence pairs with longer-than-expected tracks of 100% sequence identity (**Figure 1B**). The program was run using default parameters with the addition of the *pairwise* output option. Tracks with a global or pairwise *P*-value <0.05 were considered significant and included in follow-up analyses.

We performed a series of simulations to test for an enrichment of GENECONV tracks in repetitive elements and across several genomic contexts, including: (i) transcribed, coding, and pseudogenic regions, (ii) between pericentromeric and subtelomeric duplicons, (iii) between intra- and inter-chromosomal SDs, and (iv) between paralogs with high sequence identities. Simulations were complicated by the considerable sequence redundancy and overlap among SD families. Namely, many apparent gene conversion events localizing to identical genomic coordinates were identified in distinct SD families. Inclusion of duplicate tracks in the analysis would lead us to double count any sequence features overlapping the tracks, potentially introducing erroneous signals of enrichment. We first filtered out these duplicate tracks to obtain a set of unique, non-overlapping tracks. For a given unique gene conversion track of length *l*, we sampled one of the 1246 alignments, with sampling probabilities weighted by the total alignment length, *L*. Within the sampled alignment, two sequences were selected at random to undergo gene conversion. The start position of the simulated gene conversion track between these two aligned sequences was then drawn from a uniform distribution on (1, *L*-*l*). This procedure was repeated for each unique gene conversion track identified. A total of 1000 simulated datasets were generated, with each simulated dataset composed of a length-matched “track” for each unique gene conversion track in the actual data. We compared the observed number of tracks overlapping each tested genomic context to the distribution obtained from the 1000 simulated datasets. *P*-values were calculated as the quantile position of the observed value along the corresponding distribution of simulated values.

## Results

### Signals of Interlocus Recombination in the Human Reference Genome

We extended a previously described phylogenetic quartet method [27] to a set of 1246 high quality multiple sequence alignments from well-annotated SDs to comprehensively detect and analyze signals of interlocus recombination in the human reference genome. Briefly, we classified informative variable sites within each alignment into three sub-types: (1) C sites consistent with the dominant topology relating the paralogous sequences, (2) R sites supporting an alternative unrooted tree topology, and (3) sites of multiple mutation at paralogous positions (B sites; **Figure 1A**). If paralogs within a family have a history of interlocus recombination, the corresponding alignment may contain an excess of R sites supporting an alternative unrooted tree topology. We tested this possibility by simulating 100 sequence datasets along the tree under a model of no interlocus exchange.

**Figure 2:**
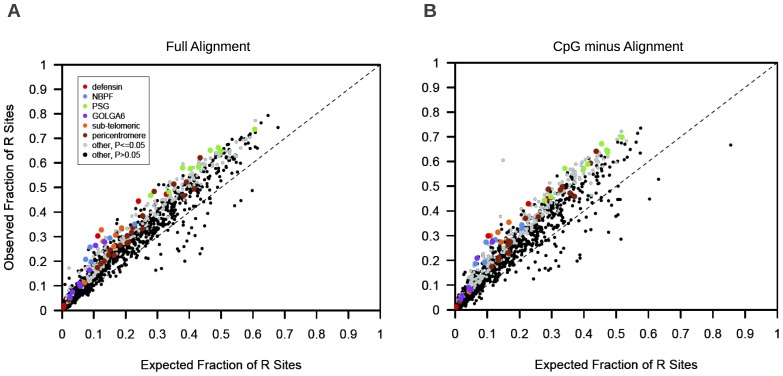
Fraction of informative sites that is reticulate. (**A**) The observed fraction of parsimony informative sites that is reticulate is plotted against the fraction of informative sites that is reticulate in simulated datasets generated under a model of no historical interlocus recombination. Each point corresponds to a single paralogous sequence family. Families with a significantly higher ratio of R sites to informative sites than predicted under the null model are shown in gray, or color-coded according to the figure legend. (**B**) The observed versus expected fraction of informative sites that is reticulate in CpG negative alignments. Black dashed line: y = x.

For each SD family, we compared the proportion of informative sites in the alignment that is reticulate to the proportion of R sites recovered in the simulated datasets. Thirty-one (2.5%) of the 1246 examined paralogous sequence families harbored no R sites and were not considered further. Of the remaining 1215 SD families, 25.4% (n = 309) had a significantly higher fraction of R sites than expected in the absence of non-allelic recombination (one-sided *P*-value ≤0.05; **Figure 2A** **, Table S2**). Strikingly, 874 families (71.9%) had more observed R sites than expected, amounting to an average 53.2% excess relative to the simulated expectation.

**Figure 3:**
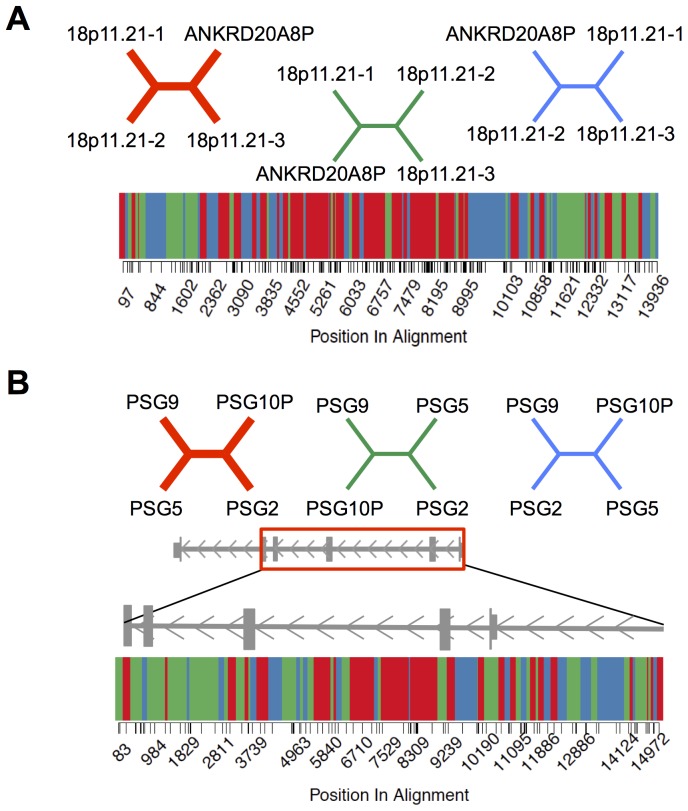
Site-to-site variation in phylogenetic history across SD alignments. (**A**) Variation in tree topology across an alignment of *ANKRD20A8P* and 3 paralogous sequences in the 18p11.21 region. The dominant topology is depicted in red, with the two alternative unrooted topologies shown in green and blue. Informative positions within the CpG negative alignment are denoted by tick marks along the x-axis. Variation in tree topology across the alignment is shown, with colors corresponding to the 3 possible topologies. The midpoint between adjacent informative sites was used to estimate positions where the tree shifts to a new topology. (**B**) Variation in tree topology across an alignment of 4 genes in the *PSG* family. The *PSG* gene model is shown, with the region corresponding to the aligned region boxed in red.

In the absence of ectopic recombination and parallel mutation (see below), each site within an SD family alignment should be concordant with the duplication history of the locus. In contrast, we observe marked site-to-site fluctuations in topology across alignments (**Figure 3**), suggesting that recombination has effectively scrambled the evolutionary history of SDs on a fine-scale. An estimated 26.1% of sites within quartet alignments lie in blocks that are inconsistent with the duplication history of the SD family, more than the simulation-based predicted percentage (18.2%; **Table S4**). This suggests that (26.1–18.2)/2 = 4% of duplicated sites in the human genome have been involved in a historical IGC event, consistent with a previous estimates from a smaller dataset [29].

### Mutation does not Create False Signals of Interlocus Recombination

In addition to non-allelic recombination, R sites can also arise by independent mutation to the same nucleotide at paralogous positions. Although such double mutation events should be rare for the closely related sequences in our dataset, we took three measures to rule out this possible explanation. First, we replicated our analysis on alignments stripped of hypermutable CpG dinucleotides. Despite a reduction in power, a comparable number of SD families still harbor a significant excess of R sites (n = 287, 24%; **Table S3,** **Figure 2B**). Second, we compared the observed fraction of B sites to the fraction of B sites in simulated datasets. B sites – sites with three different nucleotide bases represented among paralogs – necessarily arose by multiple independent mutation events at paralogous sites. None of the 1246 SD families has an excess of B sites compared with the simulated values. In most cases, significantly more B sites are expected than observed (1012 of 1246 alignments), suggesting, again, that our simulation method is conservatively biased (**Figure S1; Table S2**). Third, if R sites are largely due to stochastic mutational processes, they should be randomly distributed across alignments. In contrast, R sites that arise from interlocus recombination will tend to cluster within an alignment. We tested this hypothesis by applying a runs test [45] to the distribution of C and R sites in CpG-minus quartet alignments. Over 60% of four-paralog sub-alignments show significant under-dispersion of R sites (36,435/57,400 alignments with >5 R sites; one-sided *P*-value <0.05; **Table S4**), consistent with action of historical recombination between paralogs. Although complex mutational mechanisms can affect multiple nucleotides in a single event, these processes are overwhelmingly restricted to sites within a 10 bp window [46]. In contrast, 92% of runs of ≥2 contiguous R sites are separated by >10 bp. Taken together, these analyses indicate that the excess of R sites in our SD family alignments is not driven by mutational processes, but instead reflects historical non-allelic recombination between sequences.

### Analysis of Interlocus Gene Conversion Tracks and Crossover Breakpoints

**Figure 4:**
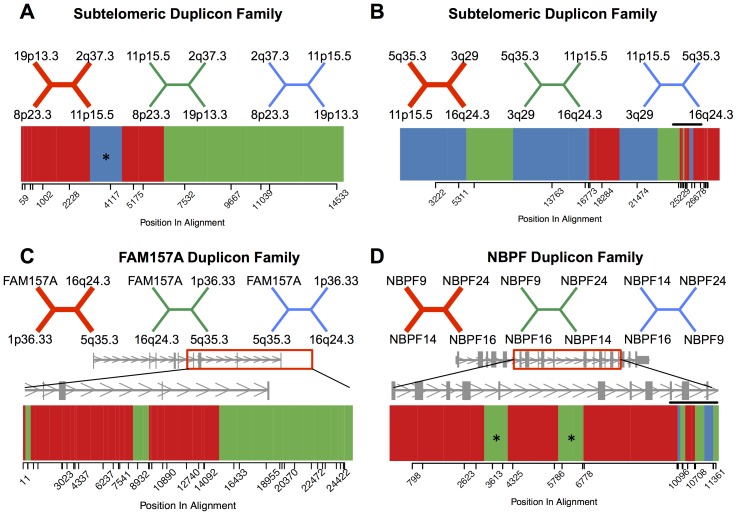
Fine-scale variation in tree topology across alignments with few predicted B sites. (**A**) The 3 possible unrooted trees relating four paralogous subtelomeric sequences on 19p13.3, 8p23.3, 11p15.5, 2q37.3 are shown. The dominant topology inferred from the alignment of these sequences is depicted in red. Informative positions within the CpG negative alignment are denoted by tick marks along the x-axis. Variation in tree topology across the alignment is shown, with colors corresponding to the 3 possible topologies. The midpoint between adjacent informative sites was used to estimate positions where the tree shifts to a new topology. There is evidence for a gene conversion event (asterisk) and an interlocus crossover breakpoint between sites 5175 and 7532 in this alignment. (**B**) Subtelomeric sequences on 5q35.3, 11p15.5 3q29, and 16q24.3. Several abrupt switches in tree topology, which could be indicative of a complex repair event, occur near end of the alignment and are denoted by a horizontal black bar. (**C**) Four members of the GUSBP gene family, with analyzed region of the consensus gene model. (**D**) Alignment of 4 sequences from the NBPF gene family. Two putative gene conversion tracks, each supported by two adjacent R sites, are indicated with asterisks. A site of potential complex repair is marked by a horizontal black bar.

We used the distribution of R sites across alignments to estimate the length of IGC tracks and the positions of interlocus crossover breakpoints. We focused on alignments stripped of hypermutable CpG sites and identified 40 SD families with at least one sequence quartet with <5 B sites predicted by simulations and >5 observed R sites (mean number of observed R sites: 9.75; mean number of B sites: 3.63; **Table S3**). Because few B sites are predicted to occur by parallel mutation events in these sequence quartets, we are confident that the majority of observed R sites arose from inter-paralog genetic exchanges, especially given that our method grossly over-predicts the number of B sites (**Table S2; Figure S1**). We identified 67 clusters of ≥1 consecutive R site(s) embedded within these quartet alignments (**Table S5;** **Figures 4A, D**). Using the midpoint between the outermost R sites in each cluster and the flanking C site on each side as the most probable start and stop coordinates of the converted sequence, we estimated a mean conversion track of 1364 bp. This track length estimate is considerably higher than estimates of gene conversion track lengths between allelic sequences [11], [13], suggesting biases toward the discovery of larger tracks in our analysis, or, possibly, fundamental differences in the mechanism of gene conversion between allelic and non-allelic sequences [16]. Notably, five of the identified conversion events have an estimated track length >3 kb, including one 9.9 kb track. On average, regions of putative gene conversion cover 11% of the sequence alignments.

We extended this strategy to identify putative crossover breakpoints between non-allelic sequences. Non-allelic crossovers will result in a switch from the dominant tree topology that extends from the crossover breakpoint to the end of the alignment, or from the start of the alignment to the breakpoint, depending on which crossover product was inherited. By excluding SD families with trees supported by <88% of bootstrap replicates, we likely eliminated most paralog pairs with historical non-allelic crossover signals. Despite this limitation of our dataset, we identified four putative ectopic crossover breakpoints, each supported by >4 consecutive R sites (**Table S6;** **Figure 4A, C**). On average, crossover breakpoints were resolved to a ∼1.4 Kb window using informative sites within each alignment (range: 561bp–2488 bp).

Our breakpoint analysis also uncovers evidence for complex interlocus exchange events characterized by several abrupt shifts in tree topology (**Figure 4** **B,D**). Such patterns could arise from multiple overlapping IGC events with variable track lengths, as might be predicted if events localize to discrete hotspots. Alternatively, clusters of tree switches could arise from single, complex repair events with DNA synthesis off multiple paralogs (*e.g.* MMBIR [47]). However, we also note that many of these apparent complex rearrangements are located at the edges of our alignments, where alignment quality is often reduced.

### Interlocus Recombination and the Evolution of Segmental Duplication Families

Our sequence quartet analysis confirms several previously reported signals of interlocus recombination in the human genome. Palindromic sequences in the male-specific region of the Y chromosome are known to undergo high rates of IGC [16], [48]. We analyzed one SD family comprised exclusively of sequences within these regions and observed a significant, 1.5-fold enrichment of R sites over the number expected (**Table S2**). Sub-telomeric repeats are also established hotspots of interchromosomal recombination [35]. We identified 15 subtelomeric duplicons with a significant excess of R sites (**Table S2**). Numerous recombination breakpoints between subtelomeric sequences were identified in analyses of site patterns across CpG negative sequence alignments, confirming these regions as hotspots of interlocus recombination (**Figure 2** **;** **Figures 4A,B** **; Tables S5, S6**). Multiple pathogenic, NAHR-mediated rearrangements have been described in the defensin gene cluster on 8p23.1, and this locus displays considerable copy number polymorphism in human populations [49]. Our analysis extends these previous findings, highlighting five SD families with a marked (1.5 to 3.8-fold) excess of R sites in this region (**Table S2;** **Figure 2**).

Beyond these classic examples of NAHR in the human genome, our analysis uncovers many novel signals. We highlight three compelling examples in the sections below.

#### *NBPF* family

The *NBPF* gene family consists of 22 genes and pseudogenes clustered in the 1p36, 1p12, and 1q21 regions. This gene family expanded during primate evolution and recent duplicates appear to be targets of adaptive evolution [3], [23], [50]. *NBPF24*, *NBPF16*, *NBPF9*, and *NBPF14* are clustered in the 1q21 region and maximum likelihood phylogenetic inference suggests that *NBPF24* and *NBPF16* duplicated most recently (**Figure 4D**). Across the CpG negative alignment for these four paralogs, there are 11 R sites, representing a 5.6-fold excess over the simulation-derived expectation (expected number of R sites = 1.96; **Table S3**). Nine of these R sites support the alternative topology that places *NBPF16* and *NBPF14* as most closely related. These nine R sites are organized into five distinct clusters, including three that are complex and possibly the result of multiple IGC events or replication-based mechanisms of repair (**Figure** **4D**). Additionally, four of these clusters are supported by ≥2 consecutive R sites, a pattern unlikely to occur by random mutation. The quartet method does not allow unambiguous determination of the sequences involved in these recombination events, but the distribution of R sites across this alignment suggests a minimum of two historical IGC events involving a combination of the *NBPF24*, *NBPF9*, *NBPF16*, and *NBPF11* paralogs, and one complex repair event. In fact, upward of half (11/22) of the informative sites across these loci may have been subject to historical interlocus exchanges (**Figure 4D**).

#### GOLGA6L family

One consequence of IGC is a reduction in inter-paralog sequence divergence, which may manifest as a decrease in the rate of molecular evolution between duplicates. We find tentative evidence of this phenomenon in the Golgin subfamily A member 6-like gene family. Paralogs in this SD family are clustered on 15q25 with an average pairwise sequence identity of 97.5%. R sites account for 54.8% of informative sites in this alignment (95% bootstrap CI: 0.43–0.67), a ∼1.7-fold excess relative to the fraction expected the absence of interlocus recombination between paralogs (38.0%; 95% CI: 24.4–50.9%). Of the 40 R sites in this alignment, 25 support a sister relationship between *GOLGA6L10* and *GOLGA6L5*. Notably, the branch lengths for these two sequences are considerably shorter than for the other two paralogs (**Figure S2**). The reduced rate of molecular evolution along the *GOLGA6L10* and *GOLGA6L5* branches could be attributable to purifying selection, but it is difficult to reconcile the excess of R sites with this hypothesis. Alternatively, frequent IGC between these two paralogs could jointly explain the observed excess of R sites and reduced branch lengths.

#### *PSG* family

The exchange of sequence variants between paralogous genomic regions can also increase haplotype diversity within loci [18]–[20], potentially accelerating the rate of adaptive evolution. Under such a scenario, paralogs are expected to display elevated nucleotide diversity and harbor a large number of shared polymorphisms. Members of the pregnancy-specific glycoprotein (*PSG*) gene family on 19q13 show strong signals of historical interlocus recombination in our analysis. These poorly characterized genes belong to the immunoglobulin superfamily and are thought to be integral to the maintenance of pregnancy [51]. Our analysis included 14 alignments with *PSG* sequences, each with a 1.03- to 2.83-fold excess of R sites compared with expectations derived from simulations (**Table S2;** **Figure 2**). Heterozygosity across the *PSG* cluster is 0.00155, nearly double the chromosome 19 average (0.00083). We used high quality SNP calls from the 1000 Genomes Project (release 20110521) [52] to identify 491 putative shared polymorphisms between *PSG* genes (**Table S7**), including 99 with minor allele frequency >0.01 in both paralogs. Average pairwise diversity between these genes is 94–95%, and incorrect read mappings may introduce spurious signals of shared polymorphism between paralogs. Nevertheless, interlocus recombination, not mutation, may account for upward of 11% of observed polymorphic variants in *PSG* genes (491 shared polymorphisms out of 4341 observed SNPs). Many genes involved in reproduction evolve rapidly [53]–[55], and we speculate that IGC could be an important mechanism of rapid adaptive evolution in the *PSG* gene family. Indeed, global pairwise alignments with chimpanzee orthologs (panTro3) indicate pairwise sequence divergences between 2.2 and 6% across transcribed portions of *PSG* genes, considerably higher than average human-chimpanzee pairwise divergence across the genome (1.2%) [56]–[58]. The *PSG* gene family could provide an exciting opportunity to study the interplay of IGC and diversifying selection, a prospect that motivates the analysis of high-quality clone-based sequences from this region in diverse human populations and non-human great ape species.

### GENECONV Analysis

Although the quartet method permits identification of SD families with an excess of R sites consistent with historical non-allelic recombination, it does not enable straightforward identification of the particular sequences driving the signal. In addition, because the method relies on the detection of sites that are discordant with the tree, interlocus exchanges between sister sequence pairs are rendered invisible. Moreover, the quartet method cannot be used to confidently resolve conversion tracks between divergent paralogs with many R sites predicted to occur by mutation. To expand our catalog of historical IGC events, we combined interlocus recombination signals from the quartet method with IGC tracks identified using a second complementary alignment-based approach that is insensitive to these limitations.

Barring subsequent mutation, sequences involved in IGC events will share complete sequence identity across conversion tracks. We used the program GENECONV to find unusually long stretches of perfect sequence identity between individual sequence pairs, conditional on the overall pattern of variable sites within the alignment (**Figure 1B**) [44]. We identified 5133 gene conversion tracks with a permutation-based *P* value <0.05. Six-hundred and sixty-six SD families (53.4%) have a least one significant track (**Table S8**), including 183 SD families that also harbor a significant excess of R sites consistent with high levels of historical interlocus recombination.

Although we observe a greater degree of consensus between GENECONV and the quartet methods than expected by chance (*P* = 0.012 by 1000 randomizations of the data), many SD families are flagged by only one of the two approaches. Indeed, only 12/67 of the putative gene conversion tracks identified in the analysis of CpG minus sequence quartets above were also identified by GENECONV. To ensure that GENECONV signals are not dominated by false positives, we used simulated sequence datasets generated in the absence of interlocus exchange as input into the program. We recovered ∼80 gene conversion tracks across a full set of 1246 simulated alignments (one per SD family; data not shown). GENECONV yields a low frequency of Type I errors in our dataset, consistent with earlier investigations on the performance of this method [29], [59]. Instead, the limited concordance between the GENECONV and quartet methods serves to emphasize differences in their statistical power under identical parameter values [36], and underscores fundamental differences in the nature of the genetic signal detected by the two approaches. In particular, because we limited our GENECONV search to conversion tracks with 100% sequence identity, our results may be biased toward the discovery of recent gene conversion tracks. The quartet method, on the other hand, may be better suited to the detection of older recombination signals that cumulatively cover a large fraction of the alignment.

Owing to the high degree of sequence overlap and redundancy among SD families, many tracks identified by GENECONV co-occur in multiple families. We filtered out these redundant tracks to obtain a final set of 1477 unique gene conversion events, corresponding to one interlocus gene conversion event per 53 kb of analyzed sequence (**Text S1**). The median conversion track length is 198 bp (mean = 385 bp) with tracks ranging in length from 3 bp to 15.0 Kb. A small percentage of tracks (1.6%; n = 24) are >5 kb, suggesting a low frequency of long track gene conversion. Collectively, tracks identified in the GENECONV analysis cover 1.54 Mb of sequence, or 4% of the genomic space surveyed in our analysis. Therefore, at least 0.77 Mb of sequence in the human reference genome has been directly converted by IGC.

### Genomic Distribution of GENECONV Tracks

Using the unique set of 1477 regions identified by GENECONV, we tested for a non-random distribution of tracks with regard to several genomic variables (see Methods). IGC tracks are preferentially located in repetitive elements, and enriched for the recombinogenic PRDM9 binding motif, CCnCCnTnnCCnC (**Table 1**) [60]. Gene conversion tracks also occur more frequently between high identity sequences located on the same chromosome, validating well-established trends (**Table 1**) [12], [28], [30]. However, despite a strong bias toward intrachromosomal gene conversion, 806 gene conversion events are interchromosomal, including numerous exchanges between subtelomeric sequences (n = 46) and pericentromeric duplicons (n = 145). Many SD families composed entirely of subtelomeric or pericentromeric sequences also show signals of interlocus recombination using the quartet method (n = 29 of 138 SD families), lending additional support to these observations.

**Table 1:** Distribution of GENECONV interlocus gene conversion tracks.

	Observed	Range of Expected Values	*P* Value
Number of pseudogene-gene events	166	146–198	0.351
Number of gene-gene events	153	140–185	0.269
Number of CDS-CDS events	22	13–32	0.393
Number of CDS-pseudogene events	34	27–51	0.301
Number of interchromosomal events	806	934–1003	<0.001
Number of interchromosomal sub-telomeric sequence exchanges	46	38–66	0.208
Number of interchromosomal peri-centromeric sequence exchanges	145	174–224	<0.001
Number of intrachromosomal events	671	474–543	<0.001
Average distance between intrachromosomal tracks	14.4 Mb	12.1–17.3	0.468
Average pairwise identity between converted sequences	96.0%	95.6–95.8	<0.001
Fraction of events overlapping SINE	0.246	0.162–0.199	<0.001
Fraction of events overlapping DNA element	0.069	0.041–0.064	0.002
Fraction of events overlapping low complexity elements	0.060	0.038–0.056	0.011
Fraction of events overlapping simple repeats	0.065	0.038–0.060	0.003
Fraction of events overlapping LTR	0.198	0.105–0.137	<0.001
Fraction of events overlapping Satellite DNA	0.038	0.017–0.032	0.003
Fraction of events overlapping LINE	0.238	0.155–0.192	<0.001
Number of PRDM9 motifs in tracks	141	48–89	<0.001

**Figure 5:**
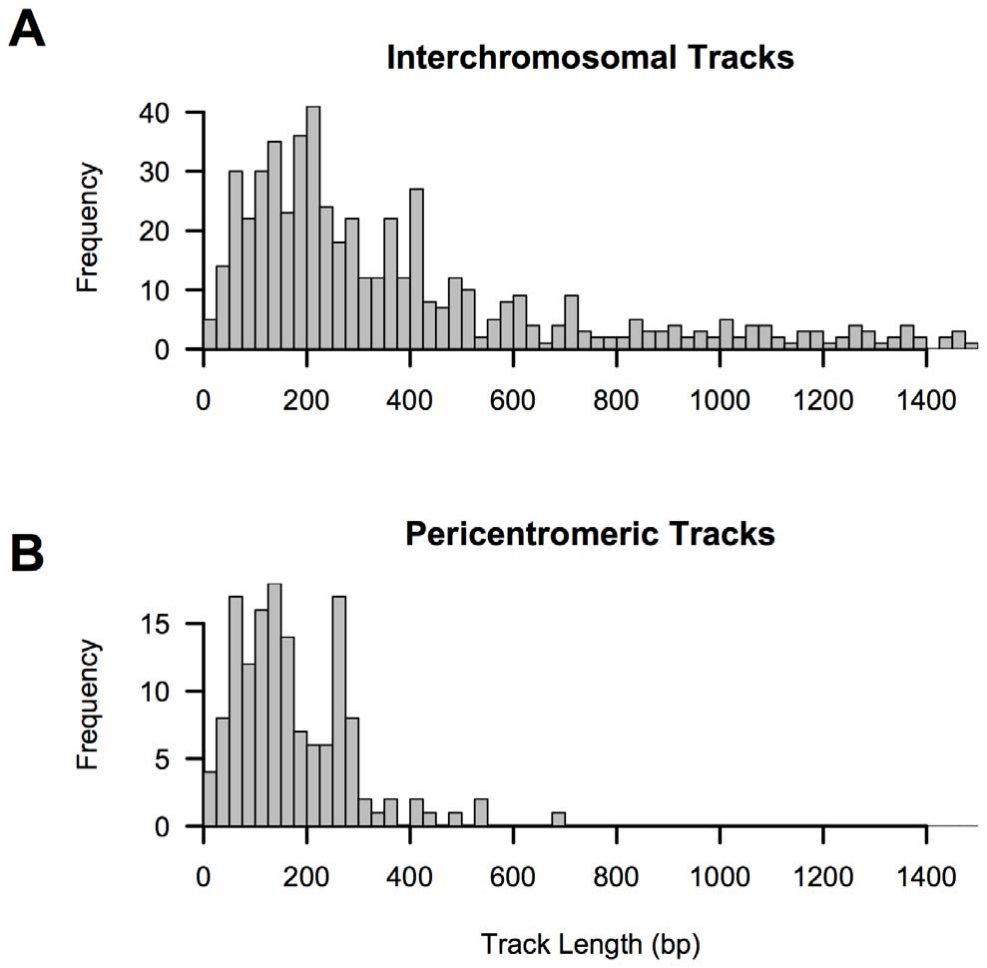
Interlocus gene conversion track lengths identified by GENECONV. (**A**) Distribution of 779 interchromosomal gene conversion tracks and (**B**) 145 interchromosomal events between pericentromeric duplicons. In order to visualize plots on the same scale, 27 tracks >1500 bp in length were excluded from (**A**).

Although we observe fewer IGC tracks in pericentromeric sequences than expected by chance (**Table 1**), the total number of conversion events (n = 145) between these regions is surprising. Meiotic crossover rates are strongly suppressed near centromeres [61], [62], and crossover events between non-homologous pericentromeric loci will result in large-scale translocations that are likely deleterious. To gain a window onto the mechanism of interchromosomal pericentromeric exchange, we analyzed the distribution of gene conversion track lengths in these regions. Strikingly, the mean length of interchromosomal pericentromeric gene conversion tracks is significantly less than the overall mean IGC track length (167 versus 585 bp; *P*<0.001 by comparison of mean track length across 1000 bootstrap replicates), as well as the average track length of interchromosomal exchange tracks (167 versus 348 bp; P<0.001; **Figure 5**). We note that pericentromeric tracks have similar flanking sequence identity to other interchromosomal gene conversion tracks identified (0.954 versus 0.956), suggesting that high local heterozygosity is unlikely triggering premature collapse of the extending D-loop during homologous strand invasion in these regions. Instead, these observations suggest differences in the mechanism of interchromosomal gene conversion across different genomic compartments. Ectopic exchanges that do occur in pericentromeric regions are strongly biased toward short track gene conversion outcomes, implying that the overall effect of NAHR in these regions in minimized.

### The Rate of IGC in the Human Genome

IGC can reduce the apparent rate of divergence between paralogs. The extent of this reduction is a function of the rate of IGC, as well as the distribution of conversion track lengths. Examination of GENECONV tracks provides an approximation to the distribution of IGC track lengths in the human genome, but direct calculation of the rate of IGC in per generation units is complicated by the fact that this mechanism violates assumptions of the molecular clock. Sequence divergence between paralogs cannot be used as a reliable metric of time since the duplication event, and independent methods of calibration, such as duplication status in closely related species, are required. With the continued emphasis on whole genome sequencing in non-human primates and other mammalian species, it may soon become possible to assign most duplication events to particular mammalian lineages, enabling more precise dating of segmental duplication and IGC rates.

These caveats aside, we can make several simplifying assumptions to derive order-of-magnitude approximations to the per generation rate of IGC in SD families. First, we assume that each paralog within a family is equally likely to be converted by any of the *n*-1 remaining paralogs in the family. Second, we ignore non-allelic crossing-over, and assume that IGC is the only mechanism of sequence transfer between duplicates. We further assume that the diversifying effects of mutation are directly counteracted by the homogenizing effects of IGC, such that the observed divergence between paralogs is at steady-state equilibrium. Under these assumptions, the per generation, per site rate of gene conversion between a given pair of paralogs in an SD family is given by:
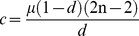
where μ is the per base per generation mutation rate, *d* is the average pairwise divergence between members of a given SD family, and *n* is the number of paralogs in the family. This result follows from equation (5) of [63].

For the SD families studied here, IGC rates range from 6.1×10^−7^ to 1.05×10^−5^ per duplicated site per generation (average: 3.2×10^−6^), assuming μ = 1.2×10^−8^ mutations/site/generation (**Table S2**) [64]–[67]. These estimates are in good agreement with previous calculations of the IGC rate in the human genome [10], [62], [68], but several orders of magnitude higher than per site rates of non-allelic crossing over [69], [70]. Our analysis surveyed 38.9 Mb (19.45 Mb duplicated sequence), suggesting that ∼60 sites are converted via gene conversion per individual per generation. In contrast, <1 *de novo* single base substitution is predicted across the duplicated sequences included in our analysis. Extrapolating these numbers to include all duplicated sites in the human genome, not just those that passed our analysis filters, yields an estimated 260 converted and two mutated sites per generation. Our calculations invoke multiple simplifying assumptions that are unlikely met in reality, and we caution against the over interpretation of these numbers. Nonetheless, it is clear that more sites in duplicated regions are converted by IGC than are targeted by *de novo* point mutation each generation.

## Discussion

We applied two complementary strategies to comprehensively catalog signals of interlocus recombination between paralogous sequences in the human reference genome (**Tables S2, S8**). In contrast to earlier studies, our analysis is not restricted to transcribed portions of the genome [28], [29], limited to regions with well-annotated orthologs in other species [29], or exclusively focused on very recently duplicated regions [27]. By expanding our analysis to all paralogous sequences >10 kb in the current reference assembly with ≥4 copies, our study presents the most comprehensive analysis of IGC in the human genome to date, surveying a total of 38.9 Mb of duplicated genomic sequence. This compilation of interlocus recombination signals provides insights into the mechanism and distribution of IGC across the human genome, as well as a community resource that will aid the interpretation of DNA diversity in segmental duplications.

We find that signals of interlocus recombination are common across duplicated sectors of the human genome. Using the quartet-based phylogenetic method, we conservatively estimate that 4% of informative sites in our SD family alignments have been targeted by non-allelic recombination. The GENECONV program uncovered 1477 unique gene conversion tracks that cumulatively cover 1.54 Mb of duplicated sequence (4% of surveyed sites). Given that few IGC tracks are identified by both methods, we conclude that between 4–8% of duplicated sites in the human genome have engaged in non-allelic recombination. Notably, this fraction is consistent with other surveys of IGC in the human genome [29], [71].

Twenty-five percent of the SD families included in our quartet analysis have a significant excess of R sites. Failure to detect an excess of R sites in the remaining 75% of SD families, however, does not exclude a role for interlocus recombination in the evolution of these paralogous sequences. Indeed, nearly all SD families have more than the predicted number of R sites, even though a minority is significant by this method (**Figure 2**). Of the 40 families with <5 B sites predicted in CpG minus alignments, only four harbor a significant excess of R sites. Nonetheless, we observe clear switches in tree topology across nearly all of these alignments, including clusters of R sites that cannot be easily explained by mutational mechanisms (**Figure 4**). Interlocus recombination may be even more frequent than results from our analyses suggest.

A previous study used the quartet method to assess interlocus exchange patterns across a set of 30 paralog families in the human genome and identified a significant excess of R sites in 28/30 tested alignments [27]. We observe a lower fraction of significant SD families in our analysis, and fail to recapitulate a significant excess of R sites in 10 of 27 SD families shared with this earlier study. We used a parameter-rich model of nucleotide substitution (GTR+I+G) for tree construction and the generation of simulated datasets. In contrast, Jackson *et al.* [27] used a F84 model of evolution [41], with no allowance for rate variation among sites. We note that estimates of the expected number of R sites are highly sensitive to the evolutionary model used to generate the simulated datasets. For example, with simulations conducted under a GTR model with no allowance for rate variation or a fraction of fixed, invariant sites, ∼85% of SD families in our dataset harbor a significant excess of R sites (data not shown). Therefore, our simulation procedure for calculating the expected number of R sites in the absence of interlocus recombination is likely conservative, and may lead to a large number of false negatives.

We suggest that many non-significant SD families in our analysis have experienced low, yet still biologically relevant, levels of interlocus recombination. In particular, if IGC tracks cluster into narrow, discrete hotspots like allelic recombination events [62], even high rates of historical IGC activity confined to narrow genomic windows could escape detection by the quartet method. Notably, we identified 483 SD families with ≥1 IGC track(s) identified by the GENECONV program, but that do not harbor an excess of R sites in the quartet analysis. A few short gene conversion tracks scattered across a large locus may not yield a sufficient number of R sites to produce a detectable signal in the quartet analysis. Instead of *P*-values calculated from alignment-wide trends, the fine-scale distribution of R sites across an alignment (*e.g.* **Figures 3** **,** **4**) may give a better pulse on the frequency and distribution of IGC in a given SD family.

### Limitations and Assumptions of Methods for Detecting Interlocus Gene Conversion

IGC is most frequent between high identity duplicons [12], [27]–[30], [72]. However, power to detect historical IGC is low when there are few distinguishing variants between paralogs [36]. Persistent interlocus exchange between highly identical sequences will further homogenize sequences, yielding an additional reduction in power. Therefore, it may be most challenging to detect footprints of IGC in regions subject to the highest rates of ectopic exchange. We note that this limitation is not specific to the quartet or GENECONV approaches used here, but a caveat of all methods for detecting IGC. Nonetheless, the GENECONV method appears to display greater sensitivity than the quartet approach for detecting IGC between high-identity paralogs [36]. Forty-one SD families in our analysis are composed of sequences with average pairwise identity >99%; none harbored a significant excess of R sites by the quartet analysis, although 24 had at least one track identified by GENECONV.

The quartet method implicitly assumes that the duplication history of the paralogous sequences in a SD family is known with complete certainty. For this reason, we limited our focus to the subset of SD families with unrooted trees supported by >88% of bootstrap replicates at all nodes. However, high rates of historical interlocus exchange will induce many topological shifts across an alignment, adding considerable uncertainty to phylogenetic inference. Therefore, our analysis may have excluded sequences with the highest rates of non-allelic recombination. At the same time, extensive IGC or interlocus crossing over could obscure the true evolutionary history of a SD family. For example, multiple gene conversion events that cumulatively cover most of an alignment could introduce an apparent sister relationship between the conversion donor and acceptor sequences that does not mirror the true duplication history of the paralogs. Although unlikely, it is possible that tree topologies from some SD families were wrongly inferred in the face of extensive interlocus exchange. However, inclusion of these SD families in the quartet analysis should not introduce false positive signals. Under this scenario, true R sites will be labeled C sites, and vice-versa, leading us to undercount the actual number of R sites.

A minimum of four sequences is required to construct an unrooted phylogenetic tree, constraining our analysis to SD families with ≥4 paralogs in the human genome. Several clinically relevant signals of IGC – such as IGC events between the *RHD* and *RHCE* genes on 1p36.11 [73]–[75] and between the von Willebrand factor gene on 12p13.31 and its pseudogene on 22q11.1 [76] – could not be confirmed using this method since they involve small paralog families. Phylogenetic quartet methods that leverage ortholog sequence comparisons (*e.g.* [29]) can be applied to families with ≥2 paralogs in each species. However, distinguishing true orthologs from homoplastic duplicates is challenging given the high rate of gene duplication and deletion. Moreover, this method is limited to the analysis of duplication events that occurred prior to speciation with the outgroup taxon, a caveat that would preclude analysis of some of the most interesting regions of recent human evolution (see discussion below). Alternatively, if the duplication history of an SD family were known, the evolutionary relationships between trios of paralogs could be rooted and the alignment analyzed for an excess of sites discordant with the rooted topology.

An additional assumption of both the quartet and GENECONV methods is that SDs have been correctly assembled into the human reference genome, with no allelic sequence variation mistaken for differences between paralogs. Most of the SD families in our analysis include sequences with average pairwise sequence identity <96%. This level of paralog sequence divergence is greater than average heterozygosity in the human genome [52], rendering it unlikely that sequences represent alternate alleles of the same locus. Moreover, most regions of segmental duplication in the human reference genome were assembled from overlapping high-quality Sanger-sequenced BAC clones, each capturing a single, unique haplotype. Nonetheless, regions of segmental duplication pose a serious challenge to *de novo* genome assembly, and errors in the human reference assembly are not uncommon (*e.g.* [26], [77]). Although we cannot firmly reject the possibility that some of the sequences included in our analysis represent allelic variants, we are confident that our dataset is overwhelmingly restricted to paralogous sequence variation.

### Mechanisms of Non-allelic Recombination

Non-allelic recombination is a general term that encompasses several distinct mechanisms of DNA repair. All forms of non-allelic recombination – including IGC, ectopic crossing over, and replication-based mechanisms for mitotic double-strand break repair such as MMBIR and replication slippage – can introduce an excess of R sites across paralog alignments. Therefore, in isolation, the quartet method cannot tease apart the relative contributions of these alternative mechanisms. Nonetheless, our survey of R site patterns in CpG minus alignments with >5 B sites provides some clues into the frequency of the various mechanisms generating R sites. Specifically, we find that 11% of aligned positions in these quartets have experienced IGC. Our quality control measures effectively eliminated sequences with interlocus crossovers, barring those with breakpoints that fall near the edges of the alignment. This point, combined with the preferential repair of meiotic double-strand breaks by non-crossover associated gene conversion [13], suggests that more R sites identified in our analysis are the result of IGC than interlocus crossing-over.

Distinguishing between IGC and complex, replication-based mechanisms of DNA repair is more challenging, especially in alignments between more divergent paralogs (*e.g.* **Figure 3**). MMBIR and replication-slippage are often associated with insertions and deletions relative to the template sequence [78]. We find that roughly one-third of R sites aggregate near gapped sites in the alignment, slightly more than expected by chance (data not shown). Although this pattern is possibly consistent with replication-based repair, gene conversion has a strong deletion bias that may also contribute to this signal [79]. Resolving the exact contributions of these mechanisms will require a more detailed analysis of sequence evolution in individual paralog families than conducted here, including comparisons with orthologous sequences. Nonetheless, meiotic double-strand breaks vastly outnumber mitotic double-strand breaks [80], leading us to favor the hypothesis that most R sites arise via IGC.

Like the quartet method, tracks reported by the GENECONV program may arise from IGC, crossovers between non-allelic sequences, or replication-based repair processes. We report that 4% of the genomic space surveyed in our analysis lies in a conversion track identified by GENECONV, implying that 2% of the duplicated sequences have been directly converted. This fraction is commensurate with a previous estimate in the human genome [29], and in-line with IGC frequency estimates from other species [81]–[83]. These observations lend further support to the notion that the majority of recombination signals detected in this analysis are due to the specific action of IGC, rather than non-allelic crossing-over or MMBIR.

### IGC and the Evolution of Human Segmental Duplications

Many SD families in our analysis have undergone recent expansions during primate evolution (**Table S2**) [30], including several with strong signals of inter-paralog recombination in our analysis. The *NBPF* and *GOLGA6L* families highlighted above, as well as the *LRRC37A*, *POM121L4*, *ANKRD20* and *SPDYE5* gene families have been focal points for recurrent duplication and transcript innovation in human-great ape evolution [84]. These “core duplicons” are inherently liable to rearrangement and ectopic recombination [85], [86], and sequences in these regions commonly show strong signals of positive selection (*e.g.* [24], [25]). The exchange of sequence variants between paralogs via IGC can have the same net effect as an increase in the local mutation rate [19]. It is tempting to speculate that IGC has directly accelerated the tempo of evolution in these regions, although this hypothesis remains untested.

The frequency of interlocus recombination in the human genome bears critically on the continued evolutionary and genetic analysis of these recent gene family expansions. First, although the duplication history of a SD family is summarized by a single tree, the evolutionary history of individual sites within the alignment may not be consistent with that topology. SD families display incredible fine-scale fluctuations in tree topology across the alignment, a pattern reflecting the local history of recombination and rare double mutation events. Second, IGC invalidates the assumption that DNA sequence divergence accrues in a clock-like fashion. The relative rates test, which is commonly used to detect departures from the molecular clock hypothesis, has little power to detect rate variation between closely related sequences [87], [88]. In the presence of IGC, branch lengths will be systematically underestimated, and estimates of duplication times based on paralog sequence divergence will introduce a systematic bias toward younger events. We urge caution when using DNA sequence data to interpret the evolutionary history of SDs in the human genome, and encourage application of orthogonal strategies, including analysis of unique, single-copy sequence immediately up- and down-stream of the duplicon, and sequence comparisons with orthologs from closely related species. Nonetheless, if IGC signals are confined to a limited number of discrete windows within an alignment, sequence-based estimates of divergence times may often be in close agreement with duplication times estimated from ortholog comparisons [71].

In addition to recently duplicated genes, our analyses also highlight the possible functional significance of IGC across segmental duplications in regions with no or minimal allelic crossing-over, such as pericentromeric regions and the Y chromosome. Regions with low recombination rates tend to show reduced DNA diversity [89] and harbor an elevated load of deleterious mutations [90]. Allelic crossovers are almost universally suppressed near centromeres, but studies in plants have suggested that allelic gene conversion may be important in these intervals [15], [91]. Rates of duplication, inversion, and translocation are elevated in centromeres [92], and our results suggest that IGC may contribute an additional layer of evolutionary dynamism in these regions. Periodic IGC between pericentromeric regions could facilitate the rapid evolution of centromeric sequences and provide a mechanism for increasing sequence diversity [93]. Similarly, high rates of IGC may help counter the otherwise irreversible accumulation of deleterious mutations across non-recombining regions of the Y chromosome [48].

Our analysis has focused exclusively on the role of interlocus recombination in the generation of fixed differences between paralogs in the human reference genome. Non-allelic crossing over is a well-established mechanism yielding copy number polymorphism in humans, but the extent to which IGC has influenced genome-wide patterns of sequence polymorphism across SDs remains untested. Our cursory analysis of shared polymorphism across genes in the *PSG* family nominates a substantial contribution of IGC to levels of segregating variation within populations. The availability of whole-genome sequence datasets from large population samples such as the 1000 Genomes Project sets the stage for more rigorous explorations of this possibility. These analyses will be complicated by non-unique sequence read placements in highly identical duplicate genomic space, which could introduce spurious signals. Despite this challenge, understanding the relationship between IGC and patterns of sequence polymorphism across SDs will shed further light on its dual roles in human disease [12], [31] and genome evolution.

## Supporting Information

Figure S1 Fraction of B sites. The observed versus expected number of B sites in each SD family alignment, expressed as a fraction of the total number of sites in the alignment. Dashed black line: y = x.
(PDF)

Figure S2 IGC and the evolution of *GOLGA6L* duplicons. (**A**) The maximum likelihood tree relating human paralogs *LOC440295*, *GOLGA6L9*, *GOLGA6L5*, and *GOLGA6L10* is shown. The value at the interior node of the tree is the fraction of bootstrap replicates (n = 100) supporting the topology. (**B**) The observed number of C sites and the number of R sites supporting the two alternate topologies are plotted, with 95% bootstrap confidence intervals (circles). Simulation derived expected values are also shown, with central 95% range of simulated values (squares).
(PDF)

Table S1 SD families excluded because of low confidence tree.
(XLS)

Table S2 Phylogenetic quartet analysis results.
(XLS)

Table S3 Results from phylogenetic quartet analysis on CpG-minus alignments.
(XLS)

Table S4 Clustering of R sites in CpG-minus sequence quartets with <5 predicted B sites.
(TXT)

Table S5 Putative gene conversion tracks identified in CpG-minus quartet alignments.
(XLS)

Table S6 Putative non-allelic crossover events in CpG-minus quartet alignments.
(XLS)

Table S7 Shared SNPs in PSG genes identified from 1000 Genomes data.
(XLS)

Table S8 Coordinates of IGC tracks identified by GENECONV.
(XLS)

Text S1 Bed format custom UCSC Genome Browser track for visualizing gene conversion events detected by GENECONV.
(TXT)
