# Supplementary figures and images for: Signals of Historical Interlocus Gene Conversion in Human Segmental Duplications
Source: PLoS One. 2013 Oct 4;8(10):e75949. doi: 10.1371/journal.pone.0075949 (PMC3790853; doi:10.1371/journal.pone.0075949)

Figure S1.

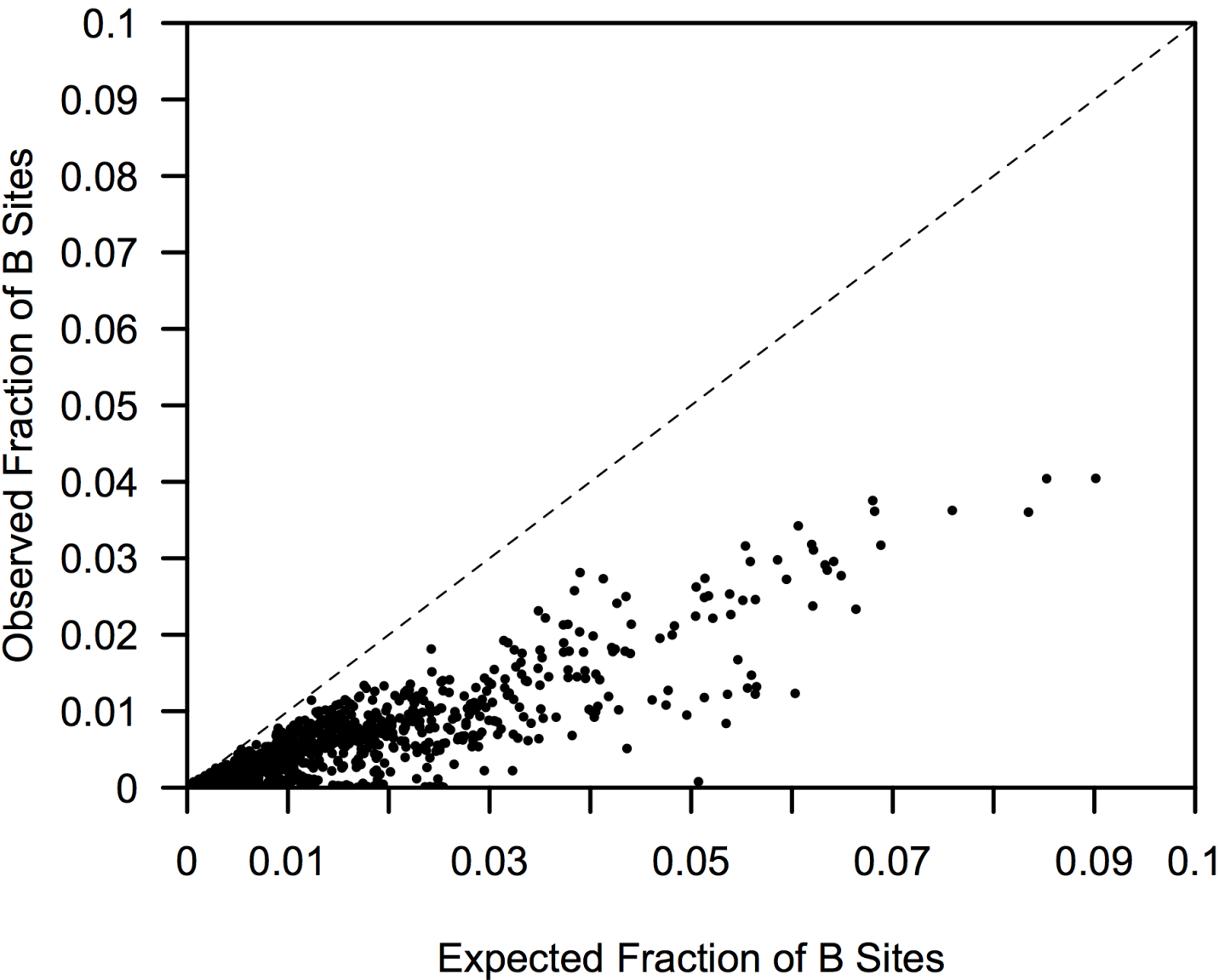

Supplement: Figure S1 — Fraction of B sites. The observed versus expected number of B sites in each SD family alignment, expressed as a fraction of the total number of sites in the alignment. Dashed black line: y = x. (PDF) [file pone.0075949.s001.pdf]

Figure S2.

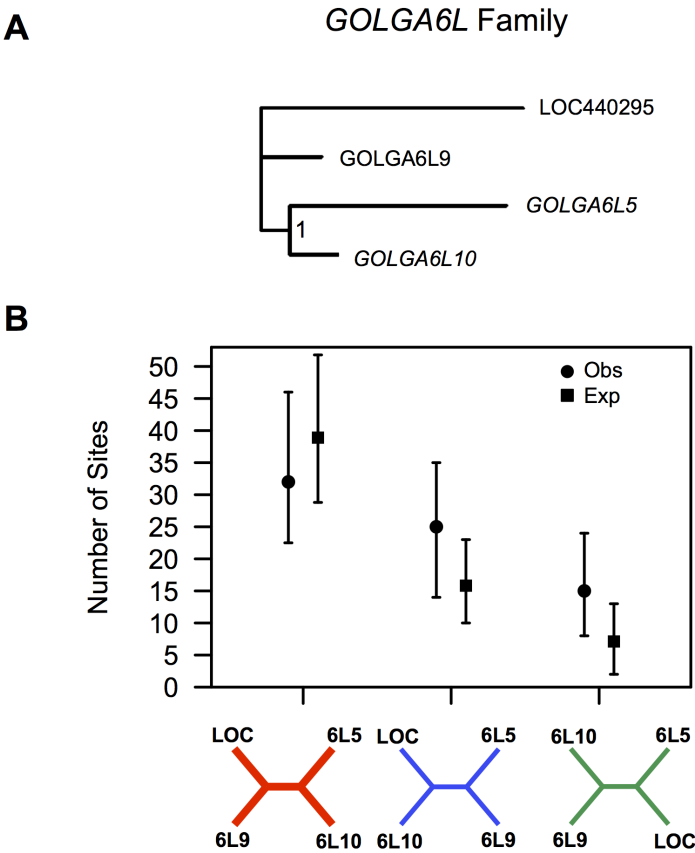

Supplement: Figure S2 — IGC and the evolution of GOLGA6L duplicons. (A) The maximum likelihood tree relating human paralogs LOC440295, GOLGA6L9, GOLGA6L5, and GOLGA6L10 is shown. The value at the interior node of the tree is the fraction of bootstrap replicates (n = 100) supporting the topology. (B) The observed number of C sites and the number of R sites supporting the two alternate topologies are plotted, with 95% bootstrap confidence intervals (circles). Simulation derived expected values are also shown, with central 95% range of simulated values (squares). (PDF) [file pone.0075949.s002.pdf]
